# Mechanism for Improving Acid-Induced Hazelnut Protein Gels Through High-Pressure Homogenization: Effect on Structural, Rheological and Gelling Properties

**DOI:** 10.3390/foods14183273

**Published:** 2025-09-21

**Authors:** Osman Gul, Abdullah Akgun, Iannie P. Maribao, Mahmut Ekrem Parlak, Furkan Turker Saricaoglu, Senay Simsek

**Affiliations:** 1 Department of Food Engineering, Faculty of Engineering and Architecture, Kastamonu University, Kastamonu 37200, Türkiye; 2 Department of Food Engineering, Faculty of Engineering, Trakya University, Edirne 22030, Türkiye; 3 College of Fisheries, Tawi-Tawi College of Technology and Oceanography, Mindanao State University, Sanga-Sanga, Bongao 7500, Tawi-Tawi, Philippines; 4 Food Engineering Department, Faculty of Engineering and Natural Sciences, Bursa Technical University, Bursa 16310, Türkiye; ekrem.parlak@btu.edu.tr (M.E.P.); furkan.saricaoglu@btu.edu.tr (F.T.S.); 5 Department of Food Science, Purdue University, West Lafayette, IN 47907, USA

**Keywords:** hazelnut protein, high-pressure homogenization, acid-induced gelation, rheology, intermolecular forces, structure

## Abstract

This study aimed to investigate the effects of high-pressure homogenization (HPH) (0, 25, 50, 100, and 150 MPa) pretreatment on the structural, rheological, and gelling properties of alkaline-extracted hazelnut protein isolate gels induced by glucono-δ-lactone (GDL). Homogenization pretreatment shortened the time required to obtain the maximum G′ value (12.65 Pa) from 32 to 28 min in the control sample. The particle size of protein isolates decreased with increasing pressure, resulting in lower particle size aggregates after gelation and in a denser gel structure with increasing gel hardness (from 1.52 g to 2.06 g) and WHC (from 31.95% to 48.36%). FT-IR spectroscopy revealed that HPH pretreatment and gelling time changed the secondary structure of the protein, promoting the formation of hazelnut protein gels. Hazelnut gel pretreated at 150 MPa exhibited the highest apparent viscosity and G′ value, indicating a more elastic and stronger gel network structure. The gel intermolecular force results showed that the contribution of hydrophobic interactions to gel formation was significant, and the chemical bond content of the gels increased with the increase in pressure up to 100 MPa. The physical stability of the gels was also improved by HPH pretreatment. Although the best WHC and physical stability were observed in the 100 MPa-pretreated gel sample, the hazelnut protein isolate pretreated at 150 MPa exhibited the best gel performance. Overall, HPH pretreatment has the potential to enhance hazelnut protein gel properties for industrial food applications.

## 1. Introduction

Protein gelation, a crucial function in food science, plays a vital role in developing the structure and texture of various food products [1] and influences characteristics such as water retention, flavor preservation, and sensory attributes [2]. The process of protein gelation involves two phases: denaturation and aggregation. While heat-induced gelation is the most common method, cold-set gels can be formed through chemical processes like enzymatic cross-linking, salt and urea addition, or acidification [3]. Recently, there has been growing interest in cold-set protein gels due to their potential as carriers for heat-sensitive bioactive components and their promising applications in food products. Glucono-δ-lactone (GDL), an acidic coagulant, can gradually lower the pH of a protein solution towards its isoelectric point by continuously releasing protons, resulting in an acid-induced gel [4]. The gentle pH reduction caused by GDL leads to the formation of gels with a uniform structure [5].

*Corylus avellana* L., commonly known as hazelnut, is a fruit with a hard shell that belongs to the Betulaceae family [6]. It holds significant economic value, with an annual yield of about 1,255,700 tons (t) in the primary producing nations, including Türkiye, Italy, USA, Azerbaijan, and Chili [7]. Hazelnuts are consumed both raw and roasted and are frequently incorporated into various food products such as confectionery, chocolate, and cakes due to their distinctive flavor and abundant bioactive compounds [8]. These nuts are abundant in major components like carbohydrates (15–18%), protein (11–16%), and oil (58–64%), as well as substantial amounts of health-promoting elements, including vitamins, minerals, organic acids, phenolic compounds, and oil-soluble bioactive substances [6,9]. The residual cake from hazelnut oil extraction serves as a valuable source of both protein and dietary fiber. Despite its high nutritional value and well-balanced essential amino acid profile, hazelnut cake is typically utilized as animal feed [10]. The protein content of hazelnut cake is approximately 35–40%, primarily consisting of two major sub-fractions: as g/100 g of protein: albumin + globulin (86.59%), glutelin (12.10%), and prolamin (1.14%) [11,12,13]. This composition makes hazelnut cake an appealing candidate for plant-based protein isolation.

Recently, incorporating proteins from plants into food products has become increasingly significant due to several factors, including affordability, environmental sustainability, health benefits, and shifting consumer preferences. Nevertheless, when compared to proteins derived from animals, those obtained from plant sources demonstrate inferior technological functionality, particularly in areas such as solubility, emulsification, foam formation, and gel-like properties [14], limiting their use as functional food ingredients in food industries [15]. As a result, researchers have developed several alternative methods for modifying plant-based protein structures that do not rely on chemical or thermal processes. These techniques, which include ultrasonic treatment, high-pressure homogenization (HPH), and micronization, aim to alter the functional properties of these proteins through structural changes [16]. HPH is particularly significant in modifying the functional characteristics of proteins and enables straightforward implementation and integration into industrial processes. It is commonly employed in the processing of natural proteins to improve their functional attributes for food manufacturing, as it minimally impacts the nutritional and flavor components of the treated food products [17]. Further benefits include its cost-effectiveness, rapid processing, and high level of efficiency [18]. HPH operates on the principle of forcing liquid through a narrow space, which accelerates its speed and results in a pressure reduction from high levels (up to 200 MPa) to atmospheric pressure (0.1 MPa). During this process, various physical effects, including high-frequency turbulence, cavitation forces, convective collision, and intense shear force, work together to alter protein structures. These changes lead to improvements in protein solubility, emulsifying and foaming capabilities, and gelation properties when used as food ingredients. The HPH process generates mechanical forces capable of disrupting both intra- and intermolecular bonds, potentially leading to alterations in the secondary, tertiary, and quaternary structures of proteins. This process can break down insoluble protein aggregates, exposing both hydrophobic and hydrophilic regions, which ultimately enhances the physicochemical and functional attributes of these protein aggregates [19,20,21].

Several researchers have studied the effects of HPH pretreatment on the cold-set gelation properties of plant-based proteins. According to Bi et al. [22], the implementation of HPH resulted in significant improvements in soy protein isolate emulsion gel properties. The storage modulus value of the gel increased from 291 Pa to 528 Pa, while its water-holding capacity rose from 87.7% to 91.4%. Additionally, the treatment led to the formation of a more stable isotropic network gel structure. Sun et al. [23] indicated that the HPH pretreatment changed the spatial structure of insoluble soybean protein isolate, significantly reduced the particle size of protein dispersion, and increased the -SH content, resulting in the formation of a stable and firm structural gel. Maribao & Gul [24] stated that HPH promotes non-covalent cross-linking between protein molecules and enhances the contact between protein particles during gelation. Accordingly, a sesame protein–gel network was formed, giving the gel a uniform and dense structure and increasing the WHC to 69.02%. Additionally, our previous study investigated the effect of HPH pretreatment on the gelation of hazelnut beverage containing 4.52% protein, 1.55% lipid, and 2.44% carbohydrate with GDL. It was observed that HPH pretreatment significantly improved the acid-induced gelation of hazelnut beverage with GDL [25]. Furthermore, it is crucial to study HPH pretreatment with hazelnut protein isolate to understand further the mechanisms involved in hazelnut protein gelation with GDL. To the best of our knowledge, the gelling behavior of hazelnut protein isolates from cold-pressed cake has not been previously reported. Furthermore, improving the gelling properties of hazelnut proteins is essential to creating gel products. This will help the food industry effectively use hazelnut protein resources. Therefore, we investigated the GDL-induced gelation of hazelnut protein isolate obtained from cold-pressed cake as a by-product of the oil industry, with the aim of investigating the improvement in gel properties by HPH pretreatment. To achieve this objective, various properties of the acid-induced hazelnut protein gel were analyzed, including its physicochemical characteristics, structural composition, rheological behavior, and gelling capabilities. Additionally, the study examined the intermolecular forces and physical stability of the gel.

## 2. Material and Methods

### 2.1. Materials

The Gursoy Hazelnut Production Factory in Ordu, Turkey provided shelled hazelnut kernels, which were subsequently skinned. A cold extraction process was employed to remove oil from the hazelnuts using laboratory-scale cold press equipment (Ecoline, NF80, Karaerler, Türkiye) at approximately 50 °C. The resulting hazelnut cake was pulverized in a laboratory blender and filtered through a 250 µm laboratory sieve to produce fine powders for protein extraction. Glucono-δ-lactone (GDL) and additional reagents were acquired from Sigma–Aldrich Co., Ltd. (St. Louis, MO, USA).

### 2.2. Hazelnut Protein Extraction and HPH Treatment

The extraction of hazelnut protein isolate was conducted using a method of alkali solubilization and acid precipitation, as previously described by Gul et al. [12]. The process began by mixing hazelnut cake powder with distilled water in a 1:12 (*w*/*w*) ratio and homogenizing the mixture using an Ultra-Turrax (Daihan, Wonju, Republic of Korea) for 3 min at 9000 rpm. The suspension’s pH was then adjusted to 10.0 using 5 M NaOH, and the resulting protein solution was stirred magnetically for 60 min at room temperature to facilitate protein transfer into the water. To separate soluble proteins from insoluble components, the suspension underwent centrifugation at 9000× *g* for 15 min at 4 °C, after which the supernatant was collected. The pH of this alkaline extract was then lowered to 4.5 (the isoelectric point) using 5 M HCl to precipitate the proteins. Another centrifugation step at 3000× *g* for 10 min at 4 °C discarded the supernatant. The resulting precipitate was dispersed in the same amount of distilled water, adjusted to pH 7.0 with 2 M NaOH and freeze-dried. The dried protein samples were ground into a powder using a laboratory grinder (Arçelik, KO9420, Ankara, Türkiye). The protein content of the final hazelnut protein isolate was determined to be 88.42% using the Kjeldahl method (N × 6.25).

To prepare the hazelnut protein isolate for HPH treatment, an 8% protein solution was created by combining the isolate with distilled water. The solution’s pH was adjusted to 7.0 using 1 M NaOH. The mixture was then agitated for 120 min with a magnetic stirrer to ensure protein dissolution, followed by overnight refrigeration. A homogenizer (Panda PLUS 2000; GEA Niro Soavi, Parma, Italy) was used to process the protein suspension at various pressures (0, 25, 50, 100, and 150 MPa) for a single cycle. These pressure levels were selected based on our previous study, which was suitable for the modification of techno-functional properties of hazelnut proteins [25]. The initial temperature of the suspension was approximately 15 °C, rising to between 17 °C and 36 °C depending on the applied pressure. The temperature of the pressurized protein suspension was tried to be kept constant with an ice-water bath. To minimize microbial growth, 0.01% sodium azide was added to the suspension before homogenization. The control sample for this study was the hazelnut protein suspension subjected to 0 MPa pressure.

### 2.3. Acid Gelation with GDL

Hazelnut protein gels induced by GDL were created utilizing 2% GDL, following the methodology outlined in a previous investigation [24]. Prior to the gelation process, the homogenized hazelnut protein suspension was brought to a temperature of 30 °C by placing it in a water bath for 15 min. Following the addition of GDL to the temperature-adjusted hazelnut protein suspensions, the mixtures were stirred at 30 °C for 5 min before being left to form gels for 1 h.

### 2.4. Acidification Kinetics

At 5 min intervals throughout the gelation procedure, the samples’ pH values were measured using a calibrated pH meter (Hanna HI 2002-02, Hanna, Vöhringen, Germany). The 180 min procedure was considered and regarded as the complete gelation period because there was no discernible change in the pH values of the gels beyond that point. The responses describing the gelation kinetics were the maximum acidification rate (*V_max_*, which is the change in the pH value (dpH/dt) with time and is expressed as an absolute value), the time to reach the maximum acidification rate (*T_max_*), the time to get pH 5.0 (*t*_5.0_), and the time to reach the isoelectric point (*t*_4.5_).

### 2.5. Particle Size and Zeta Potential

A dynamic light scattering device (Litesizer 500; Anton Paar GmbH, Graz, Austria) was employed to assess the particle size distributions of hazelnut suspensions and gel samples. The samples underwent pretreatment to ensure measurable standardization, involving ultrasound exposure at 40 KHz for 5 min, followed by vigorous vortexing for 10. Analysis of the hazelnut protein suspension and gel samples was conducted with 3–10% shading, using ultrapure water as the dispersant. The continuous phase’s refractive index was set to 1.33 for measurement purposes.

Following HPH treatment and gelation, the sample zeta potential was evaluated using dynamic light scattering (DLS) with a Zetasizer Nano ZS90 (Malvern Instruments, Worcestershire, UK) at 25 °C. The measurement process involved injecting a protein solution or gel sample (1 mg/mL) diluted with ultrapure water into the zeta cell for potential determination.

### 2.6. Fourier Transform Infrared Spectroscopy (FT-IR)

An attenuated total reflectance (ATR) diamond crystal-equipped FT-IR device (Bruker Alpha II, Karlsruhe Germany) was utilized to identify potential alterations in the chemical bond structures of hazelnut protein gels resulting from homogenization pretreatment. The protein gels were analyzed directly at 4 cm^−1^ resolution within the 4000–450 cm^−1^ wavelength range. To assess the secondary structural modifications of proteins (α-helix, β-sheet, β-turn, and random coil) caused by HPH treatment, the Amide-I band in the 1700–1600 cm^−1^ wavelength range underwent a deconvolution process with a gamma value of 2.5 and 70% smoothing length. This identical deconvolution procedure was also employed on the protein gel samples during gelation, with measurements taken at 15 min intervals over a 120 min period.

### 2.7. Intermolecular Force

The intermolecular force analysis reported by Ju et al. [26] was carried out to identify the forces that are involved in directing and sustaining gel formation, including ionic bonds, hydrophobic interactions, hydrogen bonds, and intermolecular forces between protein molecules that contribute to disulfide bonds. Four solutions were applied to the gel samples to assess the intermolecular interactions. Ionic bonds were determined using 0.6 mol/L NaCl (S1), hydrogen bonds were determined using 0.6 mol/L NaCl + 1.5 mol/L urea (S2), hydrophobic interactions were determined using 0.6 mol/L NaCl + 8 mol/L urea (S3), and disulfide bonds were determined using 0.6 mol/L NaCl + 8 mol/L urea + 0.5 mol/L β-mercaptoethanol (S4) solutions. First, 1 g of gel sample was diluted with 10 mL of S1, homogenized using Ultra-Turrax for 3 min at 5000 rpm, and then incubated for approximately 60 min at 4 °C. The mixture was centrifuged for 20 min at 4 °C at 10,000× *g*. After transferring the supernatant to a different tube and adding 10 mL of S2 to the leftover sediment, the same centrifugation, homogenization, and incubation procedures were repeated. Following centrifugation, the residual pellet was treated with S3 and the supernatant was transferred to a different tube. After centrifugation, the pellet was treated with S4, and the supernatant was transferred to a different tube. The final supernatant was collected after applying a similar procedure. The protein content of the supernatants obtained from each step was determined by the Bradford method using a bovine serum albumin (BSA) standard curve (R^2^ = 0.999). The results are expressed as BSA (mg/g) equivalents.

### 2.8. Rheological Properties

Rheological characterization of the hazelnut protein gels was conducted using a rheometer (Anton Paar, MCR302, Graz, Austria). The analysis employed parallel-plate geometry with dimensions of 35 mm in diameter and a 1 mm gap. All rheological examinations were performed at a constant 25 °C. To ensure temperature equilibrium, samples were maintained at 25 °C for 2 min before measurements commenced [25].

#### 2.8.1. Gel Formation

To assess the gelation time, GDL was introduced into the hazelnut protein suspension and mixed for 1 min using a magnetic stirrer just before conducting rheological tests. The edges of the measuring plate were coated with a thin layer of silicone oil to prevent moisture loss after transferring the GDL-containing protein solution to the rheometer plate. Each sample’s linear viscoelastic region (LVR) was evaluated using a strain sweep test at a constant frequency of 0.628 rad/s after gel formation prior to measuring the dynamic shear properties, including storage (G′) and loss modulus (G″). The gelation process of hazelnut protein solutions was monitored every 30 s using a time sweep test at 0.628 rad/s frequency and 0.1 Pa within the LVR.

#### 2.8.2. Steady Shear Tests

The flow behavior of hazelnut protein gels 1 h after the addition of GDL was investigated by measuring shear stress across a range of shear rates from 1 to 100 s^−1^. To analyze the relationship between shear rate and apparent viscosity, researchers applied the Ostwald de Waele model to the gels’ apparent viscosity data (Equation (1)).

(1) $$\eta_{app}=K\times {\gamma ˙}^{n-1}$$

where *η_app_* is the apparent viscosity (Pa · s), *K* is the consistency coefficient (Pa · sn), *γ* is the shear rate (s^−1^); and *n* is the flow behavior index.

#### 2.8.3. Dynamic Shear Tests

Dynamic rheological experiments were conducted to investigate the viscoelastic properties of hazelnut protein gels after 1 h of GDL addition. The study began with a stress sweep test at a fixed frequency of 0.681 rad/s, applying stresses from 0.01 to 10 Pa to identify the linear viscoelastic region (LVR). This was followed by a frequency sweep test within the LVR at 0.1 Pa tension, using angular frequencies ranging from 0.628 to 628 rad/s to determine the storage (G′) and loss (G″) moduli. The frequency dependence of a gel’s elastic modulus serves as an indicator of its viscoelastic behavior. To analyze the degree of frequency dependence for both storage and loss moduli in the hazelnut protein gels, researchers applied the power-law model.

(2) $$G^{\prime }=K^{\prime }\times \omega^{n^{\prime }}$$

(3) $$G^{\prime \prime }=K^{\prime \prime }\times \omega^{n^{\prime \prime }}$$

where $K^{\prime }$ and $K^{\prime \prime }$ are Power-law model constants (Pa.s^n^); $n^{\prime }$ and $n^{\prime \prime }$ are frequency exponents, and ω is the angular frequency (rad.s^−1^).

### 2.9. Water Holding Capacity (WHC)

The WHC of the hazelnut protein gels was determined according to the procedure described by Ingrassia et al. [27]. For this purpose, 20 g of the protein gel sample was transferred to a centrifuge tube and centrifuged at 2370× *g* for 5 min at 4 °C. After centrifugation, the supernatant was weighed and the WHC was calculated based on weight loss.

(4) $$WHC\left(\%\right)=\left(1-\frac{W_{1}}{W_{2}}\right)\times 100$$

where *W*_1_ and *W*_2_ are the weights of the supernatant after centrifugation (g) and the initial sample weight (g), respectively.

### 2.10. Gel Strength

The strength of hazelnut protein gels was evaluated using a TA-XT Plus Texture Analyzer (Stable Microsystems, Godalming, UK). To ensure proper gel formation and stability, the hazelnut protein suspension containing GDL was poured into a 46.1 mm diameter beaker and allowed to set for 60 min. Following this, excess surface water was removed with filter paper, and the gels were extracted from the beakers. The samples were then cut into cylindrical shapes measuring 30 mm in diameter and 20 mm in height. A cylindrical plate probe with a 35 mm diameter was employed to measure gel strength. The probe was inserted into the gel at a constant rate of 2 mm/s to a depth of 10 mm. Gel strength was determined by the maximum force recorded during the initial compression phase [25].

### 2.11. Physical Stability

The storage stability of hazelnut protein gels was evaluated by transferring 10 mL of a hazelnut protein solution containing GDL into tubes. Once the gels had matured, the tubes were kept in refrigerated conditions for a period of 15 days. Throughout the storage duration, at 2-day intervals, the liquid that had separated from the gels and accumulated on top was carefully extracted using a syringe and weighed. The degree of phase separation was then quantified as a percentage, taking into account the initial weight of the gels.

### 2.12. Statistical Analyses

The study was performed with three replications for each trial, and the results are presented as the mean ± standard deviation. One-way analysis of variance (ANOVA) was applied to the obtained data, and the differences between means were evaluated using Duncan’s test. SPSS (v22) statistical package program was used for statistical analysis, with a significance level of 0.05. The secondary structural components of the samples were quantitatively analyzed using the peak fitting approach of origin software. Origin (v18.5) software was used to create all of the figures in this study (OriginPro, Origin Lab Corporation, Northampton, MA, USA).

## 3. Results and Discussion

### 3.1. Acidification Kinetics

**Figure 1:**
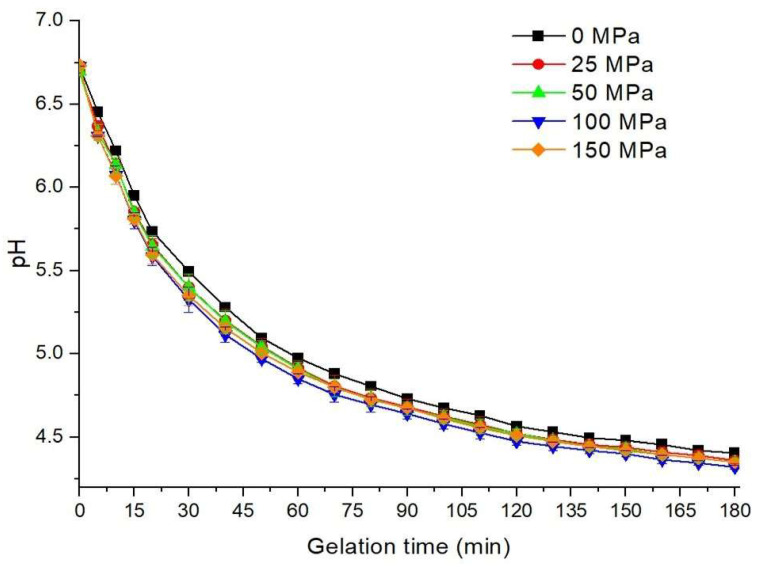
Effect of HPH pretreatment on the acidification profile of the hazelnut protein solutions induced with GDL.

GDL (2%) was added to hazelnut protein isolate suspensions (8 g/100 mL) previously subjected to different HPH pressures, and the resulting acidification profiles were monitored for 180 min. The initial pH of the suspension before homogenization was 7.02, while HPH pretreatment caused a slight decrease (0.21–0.28 units). After GDL addition, all samples exhibited a rapid pH drop within the first 5 min, reaching 6.39 in the control and 6.28–6.30 in the pretreated samples (Figure 1). Although initial pH differences were not significant (*p* > 0.05), the rate of pH decline was more pronounced in the HPH-pretreated suspensions, suggesting that homogenization facilitated faster acidification. Importantly, this effect should not be interpreted as HPH directly accelerating the chemical hydrolysis of GDL, since GDL addition occurred after homogenization. Instead, the altered acidification kinetics are more plausibly attributed to structural and physicochemical modifications in the protein suspension induced by HPH.

High-pressure homogenization is known to unfold proteins, reduce particle size, and increase molecular dispersion, leading to enhanced exposure of ionizable groups [25,28]. These structural changes may reduce the buffering capacity of the hazelnut protein suspension, such that the same amount of gluconic acid released from GDL hydrolysis results in a faster measurable pH decrease. In addition, HPH improves the homogeneity of the system, ensuring rapid distribution of GDL and its hydrolysis products. Consequently, the observed acceleration in the early acidification phase likely reflects protein-related modifications rather than direct catalytic effects on GDL dissociation. As shown in Figure 1, while the pH change in the samples is faster at the beginning of the gelation period, the pH change tends to slow down as time progresses. Similar results were observed by Maribao & Gul [24], Gul et al. [25], and Pang et al. [29], and it was reported that a rapid decrease in the pH value occurs in the first minutes with the hydrolysis of GDL to gluconic acid, and then the pH decreases steadily. At the end of the gelation period, the pH value of the control sample was 4.41, while it was determined between 4.32 and 4.36 in the HPH-pretreated samples, and the lowest pH was observed in the sample homogenized at 100 MPa pressure.

The acidification kinetic results of hazelnut protein suspension samples are given in Table 1. Acidification kinetics were evaluated by considering the maximum acidification rate (*V_max_*), time to reach *V_max_* (*T_max_*), time to reach pH 5.0 (*T*_5.0_), and time to reach pH 4.5 (*T*_4.5_). HPH-pretreatment affected the change in the pH value of the hazelnut protein suspension after the addition of GDL, and the *V_max_* value tended to increase depending on the increase in the homogenization pressure, and the highest value was determined in the sample pretreated at 150 MPa. Although *V_max_* values of the pressured samples differed numerically, no statistically significant difference was detected (*p* > 0.05). However, the *V_max_* value in the sample pressured at 150 MPa was 0.09 pH unit/min, which was significantly higher compared to the control sample (*V_max_* = 0.066 pH unit/min) (*p* < 0.05). The increase in the *V_max_* value with increasing pressure is probably due to the faster dissociation of GDL due to the change in dissociation constants of acids and bases during homogenization.

The *T_max_* value was determined to be 5 min in all samples, and there was no difference. On the other hand, differences were detected in the times for the samples to reach pH values of 5 and 4.5. While the control sample required 60 min to reach pH 5, it was determined as 52.5 min in those applied at 25 and 50 MPa pressures and 50 min at 100 and 150 MPa pressures. The time for the pH value to reach the isoelectric point (4.5) was determined as 142.5 min for the control sample, and it decreased with the increase in the applied pressure, and the lowest value was determined in the sample pretreated at 100 MPa. The *T*_4.5_ value increased partially when the pressure was increased to 150 MPa. In the study conducted by Maribao & Gul [24], it was reported that the time required for the pH value of the control sample to reach the isoelectric point was 150 min, and this time decreased to 100 min with the application of 100 MPa pressure. On the other hand, there are also findings in the literature that HPH-treated soy flour suspension did not affect the pH change during gelation [28].

### 3.2. Gelation Kinetics

**Figure 2:**
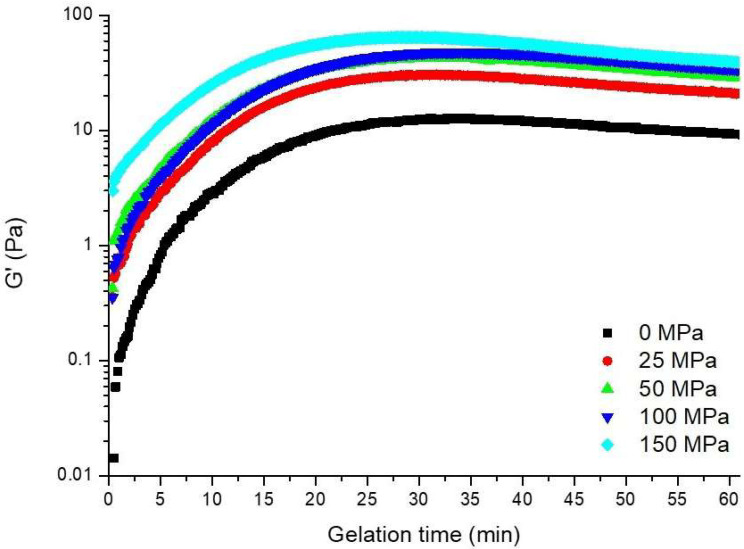
Gelation kinetic of the acid-induced hazelnut protein isolate gels produced from HPH treated hazelnut protein solution.

The effects of homogenization pretreatment on the acid gelation of hazelnut protein isolate were examined by monitoring changes in the elastic modulus (G′) after GDL addition (Figure 2). G′, which is linked to the elastic properties of the gel network during formation, quantifies the material’s ability to store elastic deformation energy in each dynamic oscillation cycle. This measurement provides insight into the material’s elastic or solid characteristics, reflecting its structural properties during the gelation process [30]. In all samples, a rapid increase in the G′ value was observed in the early stages of gelation (in the first 15 min) after the addition of GDL, indicating that the system made a very rapid transition from a fluid to a viscoelastic solid [31]. Subsequently, the rate of increase slowed and became stable. When Figure 2 is evaluated visually, no significant difference is detected in the time required to reach the maximum G′ value. However, when examined numerically, 32 min was required to obtain the maximum G′ value (12.65 Pa) in the control sample; this time was reduced to 28 min with homogenization treatment. After attaining the peak G′ value, the samples stabilized, indicating the establishment of a durable acid-induced gel structure. This structure resulted from protein aggregation occurring at a pH near the protein’s isoelectric point, triggered by the liberation of hydrogen protons during GDL-mediated acidification [32]. A similar trend in the gelation of plant proteins has been observed by different researchers [22,25,33,34], which has been reported that the rate of increase in the G′ value is high due to the high dissociation rate of GDL at the beginning of gelation and then it reaches a stable state (15–20 min). The observed gelation kinetics are typical of biopolymer gelation, and the higher the G′ value expressed, the stronger the intermolecular network and the higher the interaction between proteins [35].

As shown in Figure 2, although the gel formation times of the samples were similar, the elastic properties of the hazelnut protein gel became stronger as the applied homogenization pressure increased, and the hazelnut protein gel became a more rigid system with homogenization pretreatment [36]. A similar result was obtained for Huang et al. [34] for the high-pressure homogenization applied soy protein isolate gel. This is probably related to the fact that homogenization pretreatment can destroy the covalent cross-linking of disulfide bonds in the stereoscopic network structure of the protein and accelerate the expansion of protein groups. Thus, it can lead to better protein network interactions, reduce the volume of the network, and improve the contact surfaces [22,35]. The application of HPH causes a decrease in particle size and increased solubility of hazelnut protein [11], thus leading to neutralization of the surface charges of soluble aggregates. Consequently, the van der Waals attraction and hydrophobic interactions of these neutralized aggregates become dominant and trigger coagulation [37,38]. This contributes to the formation of more homogeneous and robust gel networks.

### 3.3. Particle Size Distribution and Zeta Potential

**Figure 3:**
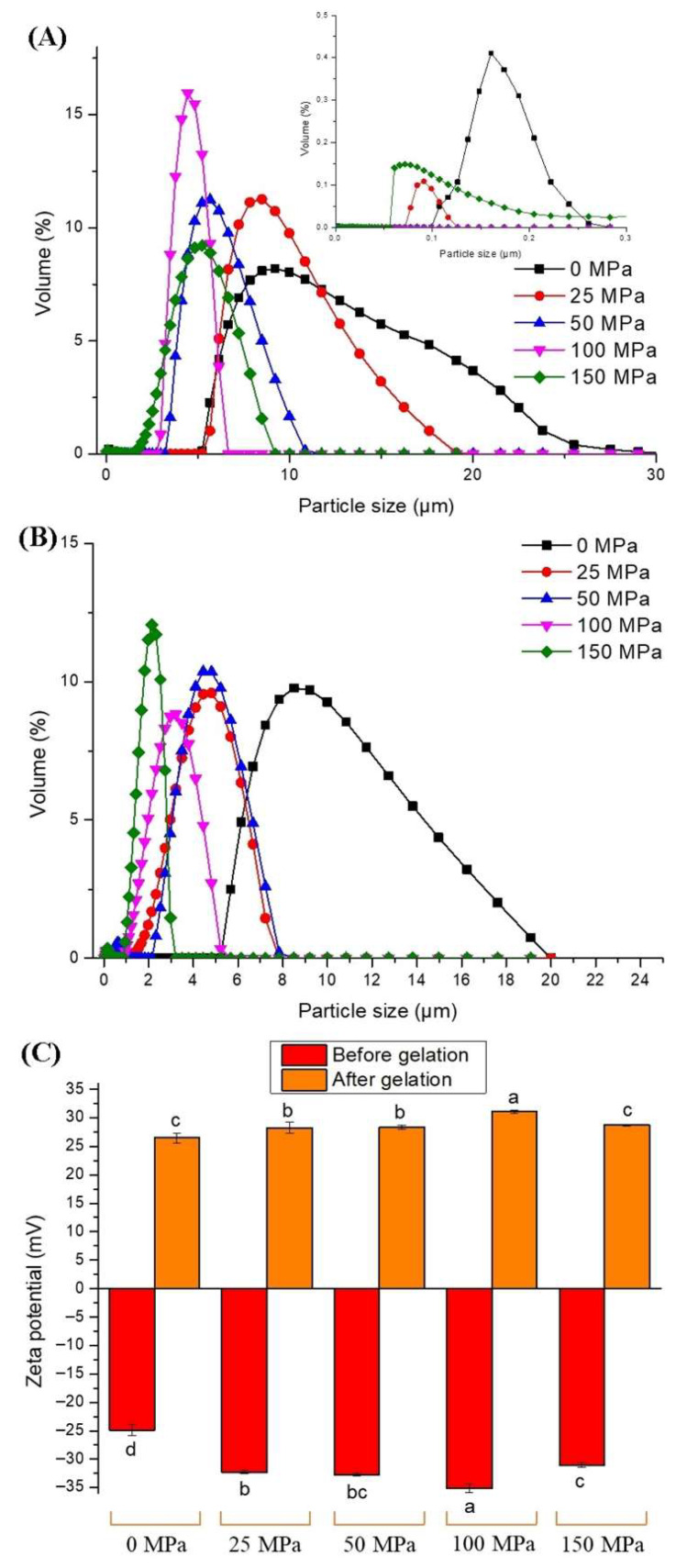
Particle size distribution of samples before (**A**) and after (**B**) the gelation. Zeta potential (**C**) of the hazelnut protein solutions and gels. The lowercase letters on the bar graph represent significant differences between the samples (*p* < 0.05).

The change in the particle size distribution due to HPH treatment at different pressures on the hazelnut protein suspension is shown in Figure 3A. HPH treatment significantly altered the particle size distribution of hazelnut protein suspensions. A bimodal distribution was observed in the control, 25 MPa, and 150 MPa pressure-applied samples, with one peak in the smaller particle size region (<0.3 μm) and another peak at approximately 10 μm. In contrast, a unimodal distribution was detected in the other samples. With an increase in homogenization pressure, the particle size distribution shifted to a smaller particle size region (5 μm). The distribution range of the control sample was wider (5–29 μm) than that of the other samples, and the particle diameter distribution was in a narrower range with the homogenization application, indicating a more homogeneous distribution. On the other hand, at extremely high pressure (150 MPa), a bimodal distribution was observed in the protein suspension, which is probably related to protein aggregation and denaturation due to high pressure [11]. Ma et al. [39] reported that the main peak at 1000 nm disappeared, and the maximum peak position shifted towards the smaller particle size range (100–300 nm) with the increase in homogenization pressure, and the samples showed a narrower unimodal distribution.

The HPH treatment of the hazelnut protein suspension before gelation also caused the particle size distribution of the protein gels to differ (Figure 3B). The particle size distribution in all gel samples was unimodal. The highest and widest distribution range (5–20 µm) was observed in the control gel sample. The HPH pretreatment shifted the particle size distribution of the gels to the left and caused them to show a narrower range with a more homogeneous distribution. As particle size is reduced through high-pressure homogenization (HPH), protein solubility increases [11]. Consequently, the enhanced solubility of proteins leads to the formation of macroscopic gels with a more uniform structure [40].

Zeta potential determines the distribution and aggregation of suspended particles by reflecting the charge status on the surface of the particles [41]. The zeta potential values of the protein gel obtained after homogenization at different pressures and by adding GDL to the hazelnut protein suspension are shown in Figure 3C. The hazelnut protein suspensions exhibited negative zeta potential values, suggesting that the protein surface contained a higher proportion of negatively charged amino acids compared to positively charged ones [42]. The zeta potential value was measured as −24.9 mV in the control sample, and the zeta potential value showed a significant difference depending on the applied homogenization pressure (*p* < 0.05). At homogenization pressures up to 100 MPa, the absolute zeta potential of the protein suspension tended to increase and reached a maximum of −35.15 mV. A decrease in the potential value was observed with higher pressure application (*p* < 0.05). Similar results were obtained in our previous study on hazelnut protein isolate [11] and in studies conducted with other plant protein isolates in the literature [16,17]. The fact that the absolute zeta potential in the control sample was lower than that in the homogenized samples indicates that the electrostatic repulsion between the protein particles was weak. However, the partial unfolding of proteins subjected to high-pressure homogenization and the exposure of charged amino acids on the protein surface played a role in the increase in the absolute zeta potential [17]. The decrease in absolute zeta potential indicates that the proteins aggregate at extremely high pressures [43]. HPH pre-treatment at 150 MPa resulted in a decreased absolute zeta potential, and this could be related to a higher particle size than 100 MPa treatment, showing the partial aggregation of protein particles. Ma et al. [39] reported that the zeta potential of the chickpea protein emulsion increased with an increase in homogenization pressure, and the absolute value reached a maximum at 60 MPa (two passes) and 90 MPa (one and three passes), while the zeta potential decreased at higher pressures. Zeta potential values showed significant differences owing to the decrease in pH with the addition of GDL. When the pH value decreased below the isoelectric point of the protein, the surface charges of the proteins became positive, and the positive charge increased as the pH decreased (Figure 3B). The zeta potential values of the gels were consistent with the pH values after gelation, and the lowest and highest zeta potential values were measured in the control and 100 MPa pressure-applied samples with the highest and lowest pH, respectively. A low zeta potential value indicates that the electrostatic repulsion is weak, and the decreased electrostatic repulsion indicates that protein molecules can easily form a gel and thus achieve a higher gel hardness [5]. In our study, the zeta potential was measured after the completion of gelation and was clearly related to the pH of the samples at the end of gelation. A higher zeta potential value indicates a higher energy barrier between the particles, which reveals stronger electrostatic repulsion and a more stable colloidal system.

### 3.4. FT-IR

**Figure 4:**
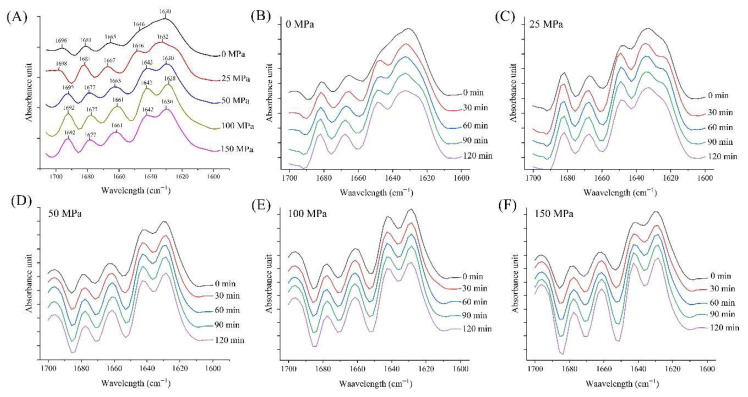
FT-IR spectra and related peaks of the acid-induced hazelnut protein isolate gels produced from HPH treated hazelnut protein solution ((**A**): FT-IR spectra in the Amide I region for all samples; (**B**–**F**): the change in FT-IR spectra in the Amide I region during gelation period for 0, 25, 50, 100 and 150 MPa treated hazelnut protein samples, respectively).

The FT-IR spectra in the Amide I region (1600–1700 cm^−1^), as depicted in Figure 4, revealed secondary structural changes in hazelnut proteins under varying pressure conditions and gelation times. The Amide I band is sensitive to the secondary structure of the protein, such as α-helices, β-sheets, β-turns, and random coils. Shifts in peak positions and intensity provide insights into protein conformational changes during cold-set gelation with glucono delta lactone (GDL) and pretreatment with high-pressure homogenization (HPH).

**Figure 5:**
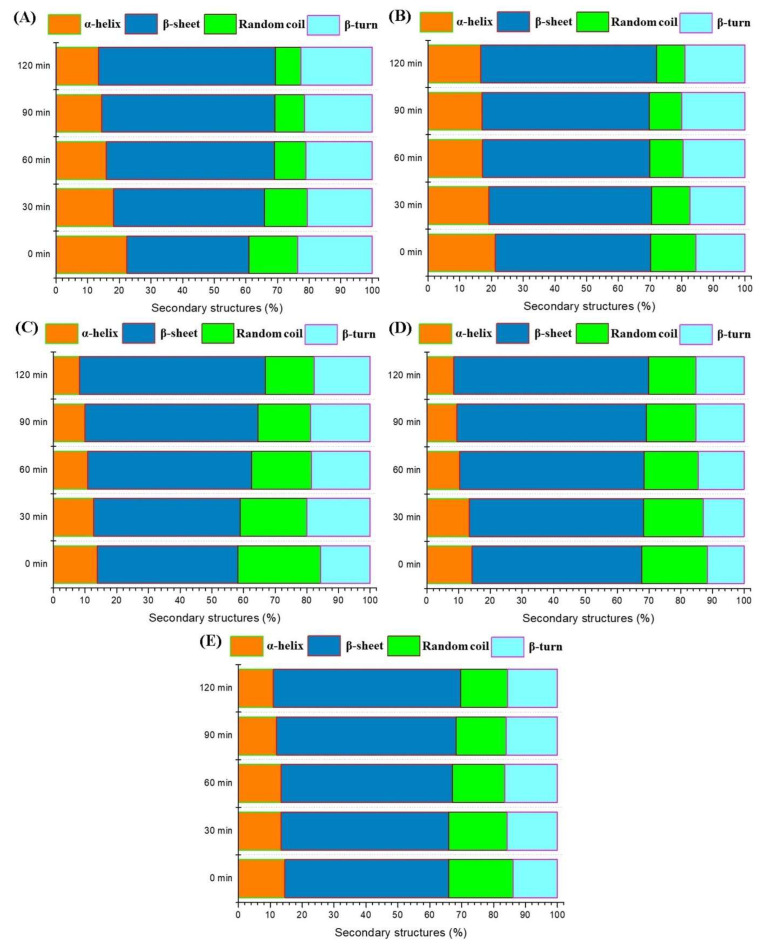
Change the secondary structure (%) derived from deconvolution of amide I band of the hazelnut protein isolate gels during gelation ((**A**): 0 MPa, (**B**): 25 MPa, (**C**): 50 MPa, (**D**): 100 MPa, (**E**): 150 MPa).

In the leftmost plot, the spectra show that increasing pressure (from 0 to 150 MPa) affected the secondary structure of hazelnut proteins (Figure 4A). With increasing pressure, notable shifts and changes in the peak intensities of the Amide I band were observed. For instance, peaks around 1630–1640 cm^−1^, typically associated with β-sheet structures, became more prominent under higher pressure, and an increase in β-sheet content was crucial for the formation of protein aggregates (Figure 5). Ni et al. [44] stated that the generation of β-sheet content promotes the formation of myosin gel, and Choi & Ma [45] also stated that the β-sheet structure has a beneficial effect on the formation of protein gels. Conversely, peaks in the 1650–1660 cm^−1^ range, indicative of α-helices, were not observed in the 0 MPa-pretreated sample and decreased in intensity with pressure, reflecting a loss of α-helical structures.

Time-dependent plots (0–120 min) further highlighted the dynamic structural rearrangements of the proteins. At higher pressures, such as 100 and 150 MPa (Figure 4E,F), the time-dependent changes were more pronounced. Over time, the secondary structure of the proteins evolved from predominantly α-helical or random coil conformations to more β-sheet structures as the gel matured. This transition is often accompanied by an increase in the gel’s elasticity and viscosity, indicating a more robust network formation [46,47]. HPH pretreatment enhanced the exposure of hydrophobic and reactive sites, facilitating intermolecular interactions (protein–protein interactions) and network formation during gelation. Peaks around 1630 cm^−1^ were linked to β-sheets, which were critical for gel stability. Peaks near 1650–1660 cm^−1^ represented α-helices, while peaks around 1680–1690 cm^−1^ were associated with β-turns or aggregated structures. The observed increase in β-sheet structures under high pressure and over time showed a structural transition that favored gelation. When combined with GDL, which reduced pH to promote protein aggregation, the structural changes induced by HPH supported a stronger and more stable gel network. The transition from α-helices to β-sheets aligns with the mechanism of gel formation under acidic conditions, where β-sheets play a dominant role in network assembly. This could also be related to the decreasing particle size and increasing surface area of proteins by HPH pretreatment, leading to a more stable gel network [25]. The contents of α-helices and β-sheets were strongly correlated with the gel strength and water retention of protein gels [48]. In this study, the impact of HPH pretreatment on the α-helix and β-sheet content in the gel samples was in line with the WHC and gel strength.

### 3.5. Intermolecular Forces

As protein gels form, the proteins develop new intermolecular interactions and potentially undergo structural modifications, resulting in an organized aggregation that creates a three-dimensional network structure. These interaction forces between protein molecules lead to the gel reaching equilibrium [49,50]. Intermolecular forces are key indices affecting the properties of protein gels, and they are also correlated with the mechanical properties of gels. Through superposition treatment with various solvents (S1, S2, S3, and S4), the relative proportions of ionic bonds, hydrogen bonds, hydrophobic interactions, and disulfide bonds in hazelnut protein gels were ascertained. The formation of protein gels primarily occurs through the interconnection of polypeptide chains, which helps to maintain the gel’s structure [49]. Additionally, research has shown that hydrophobic interactions, disulfide bonds, and hydrogen bonds contribute significantly to the coagulation process during protein cross-linking within the gel system [50]. As can be seen from Table 2, the order of different bond types was the same in all samples and was as follows: hydrophobic interaction > disulfide bond > hydrogen bond > ionic bond. The findings suggest that hydrophobic interactions play a crucial role in the formation of gels from hazelnut proteins. Similarly, it has been reported that hydrophobic interactions and disulfide bonds are dominant in the GDL-induced gelation of sesame protein [24] and soy protein subunits [26], and the contribution of ionic and hydrogen bonds is relatively low. Ionic and hydrogen bonds are mainly found between the polar groups in protein molecules [51]. In these gels composed of subunits or polypeptides, the electrostatic forces that cause repulsion among the protein molecules’ polar groups are reduced as GDL hydrolysis releases H+ ions [52]. Thus, the ionic and hydrogen bond contents were low in all gel samples. On the other hand, hydrogen bonds have a low contribution to gel formation but play an important role in the water retention capacity.

The application of homogenization to hazelnut protein suspensions at different pressures significantly affected the bond distribution during gelation with the GDL (*p* < 0.05). It was determined that the dominant chemical bond content of the gels increased with increasing applied pressure and decreased at extremely high pressure (150 MPa). This change due to homogenization pretreatment is compatible with previous studies [53]. According to Zhang et al. [43], the increase in the content of all chemical bonds with increasing pressure can be explained by the partial denaturation of protein molecules occurring during homogenization. Partial denaturation can lead to opening of the protein, exposure of hydrophobic residues and internal -SH groups, and increased intermolecular interactions. Thus, the hydrophobicity of the protein increased and disulfide bonds were formed. However, protein structures can be disrupted by physical factors (such as high shear force and cavitation) that occur during homogenization. When the pressure in homogenization is elevated, it leads to the formation of smaller particles, increased particle density, enhanced Brownian motion, and more frequent particle interactions. This can result in a higher degree of non-covalent protein cross-linking within the suspension as it undergoes high-speed flow in the homogenizer. Additionally, alterations in secondary structures (α-helix) may cause protein rearrangement, further contributing to protein cross-linking. This process plays a crucial role in the formation of protein gels, resulting in a uniform and compact gel structure with improved water holding capacity (WHC) [53,54].

Denaturation and aggregate formation in proteins owing to extreme HPH pressure can explain the decrease in bond structures. These results are consistent with the WHC results; the decrease in WHC resulting from extremely high pressure application may be attributed to a reduction in hydrogen bonding. According to research by Ju et al. [26], despite their relatively small contribution, hydrogen bonds are crucial for maintaining the water-retention properties of gel samples derived from soy protein subfractions.

### 3.6. Rheological Behavior

#### 3.6.1. Apparent Viscosity

**Figure 6:**
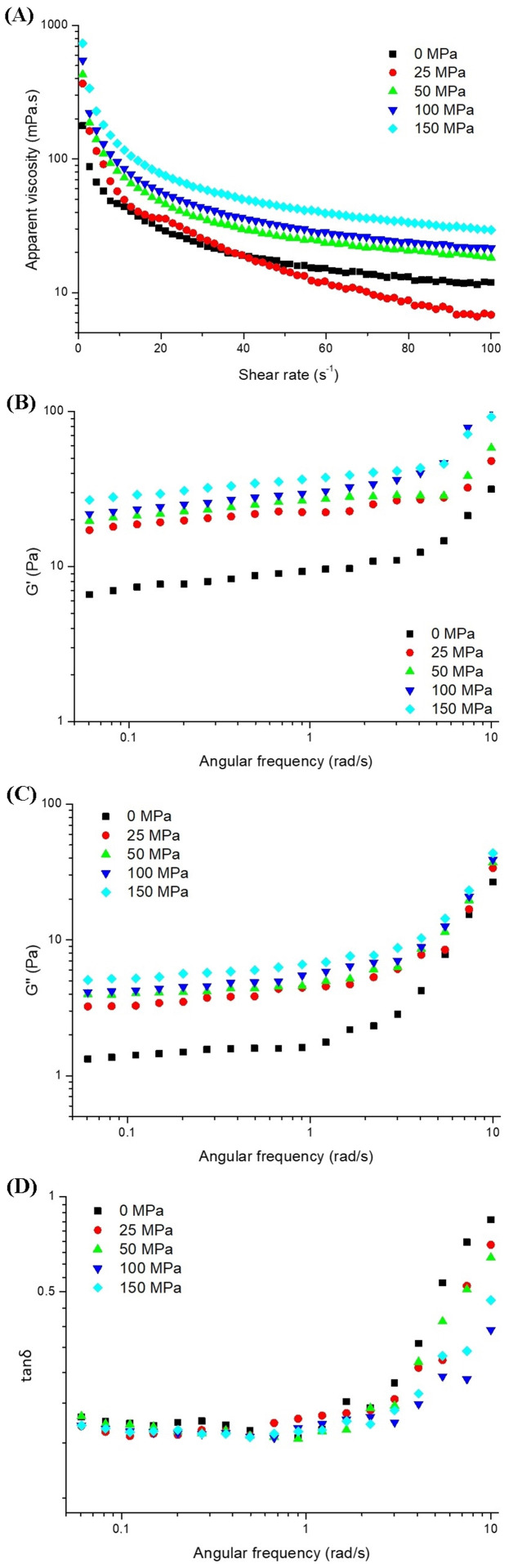
Apparent viscosity (**A**), storage modulus (**B**), loss modulus (**C**) and tanδ (**D**) of the acid-induced hazelnut protein isolate gels produced from HPH treated hazelnut protein solution.

The flow properties of hazelnut protein gels with and without homogenization pretreatment are shown in Figure 6A. The apparent viscosity (η) in all samples tended to decrease with increasing shear rate, and they exhibited non-Newtonian pseudoplastic flow (shear thinning) behavior, which is typical for semi-solid systems. Similar flow behavior was observed for sesame protein gel [24] and hazelnut beverage gel [25]. According to McClements [55], shear thinning behavior is associated with the disruption of the particles or gel network that have come together with protein-protein or protein-water interactions at high shear forces, which causes a decrease in apparent viscosity. The rate of decline in the apparent viscosity of the samples along the shear rate varied depending on the homogenization pressure. Generally, the control samples and those treated at 25 MPa exhibited a swift decrease in apparent viscosity up to a shear rate of 10 s^−1^, after which the values stabilized at higher rates. Conversely, gel samples subjected to pressures of 50 MPa and above displayed a rapid viscosity reduction initially (at a 5 s^−1^ shear rate), followed by a gradual decrease before stabilizing. This behavior indicates that the homogenization pretreatment and applied pressure-induced structural modifications in the hazelnut protein gel [25,56].

Considering the viscosity values of the samples, while the lowest viscosity value was determined in the control sample at the beginning, the viscosity of the sample pretreated at 25 MPa was determined to be the weakest at the end of the analysis because of the higher rate of viscosity decrease. In contrast, the highest apparent viscosity value during the study (at shear rates of 1–100 s^−1^) was found in the hazelnut gel sample pretreated at 150 MPa. Homogenization pretreatment (except for 25 MPa) caused an increase in the apparent viscosity of the hazelnut gels. Similar results were also observed by Maribao & Gul [24] for sesame protein gel, Ge et al. [57] for pork myfibrillar protein with HPH-modified soy 11S globulin, and Xie et al. [53] for cod protein gel. With an increase in homogenization pressure, a decrease in the size of the protein particles and an increase in the specific surface area occur [53]. The increase in the specific surface area (contact area between particles) promoted cross-linking between protein molecules, which increased the apparent viscosity of the protein gel with cross-links between proteins. Likewise, Alvarez-Sabatel et al. [58] reported that the application of high pressure could enhance the particle-specific area, leading to improved water retention capabilities of inulin. This, in turn, contributes to better development of the gel network structure.

The apparent viscosity and shear rate relationship of the hazelnut protein gels was well-fitted to the Ostwald-de-Waele model. The model parameters, as well as the apparent viscosity values at a specific shear rate (50 s^−1^), are listed in Table 3. The flow curves of gel samples fit well with the Ostwald-de-Waele model due to the high coefficient of determination (*R*^2^ = 0.9915–0.9975). The *n* value was determined to be between 0 and 1 for all gel samples, which can be considered pseudoplastic fluids. Similar results were observed in samples of hazelnut beverage gel [25] and sesame protein gels [24]. A decrease in the *n* values of the protein gels occurred because of an increase in pressure. However, an increased *K* value was observed in the gels owing to the increased pressure. The sample subjected to pretreatment at 150 MPa exhibited the maximum K value, suggesting that the gel structure formed under these conditions was more robust compared to other samples.

#### 3.6.2. Frequency Sweep

Oscillation (amplitude vibration) tests were performed to obtain information about the elasticity of the food materials. Therefore, a frequency sweep test was carried out to determine the viscoelastic properties of hazelnut protein gels, and the results are shown in Figure 6B–D. All samples exhibited similar shapes in their frequency sweep patterns, with G′ and G″ values showing an increase as frequency rose. The way G′ and G″ values changed with frequency was consistent with earlier findings for various hydrocolloidal protein gel types [24,59]. The region where G′ and G″ values rise with increasing frequency is termed a plateau zone, situated in the mechanical spectrum between the terminal and transition areas. This intermediate section is characterized by the upward trend of these values as frequency escalates [60]. The increase in the G′ value in all frequency sweeps was observed to be higher than the G′ value for all samples, indicating the formation of a continuous network structure [61]. A network-like structure (G′ > G″) at all frequencies has been reported in the literature for acid-based peanut protein isolate gels [62], whey protein emulsion gels [63], and sesame protein gels [24].

As shown in Figure 6B,C, the storage and loss moduli of the gels tended to increase depending on the applied homogenization pressure, and the elastic dominant gel-like behavior of the samples was found to be dominant in the linear viscoelastic region. This shows that when the homogenization pressure was high, the hazelnut protein gel was more elastic and the network structure of the gel was stronger. A similar result was observed by Sun et al. [23], who reported that the G′ and G″ values of acid-derived gel samples prepared using soy protein solution pretreated with HPH gradually increased with increasing pressure. Kang et al. [64] found that the G′ and G″ values of the soy 11S protein gel at the same frequency increased significantly with increasing pressure, and the G′ value was more significant than the G″ value. In another study conducted by Bi et al. [36], it was determined that the storage modulus was higher than that of the untreated sample, owing to the high-speed shear applied to the soy protein isolate. This showed that the protein gels obtained after the HPH process were more stable and had a stronger gel structure than the control sample. It is thought that the decrease in particle size and increase in protein solubility of protein isolates after HPH application are practical for forming a stronger gel structure. Increased protein solubility and the exposure of more protein molecules to acidification may be factors in improving the gelation properties of the protein [65]. In addition, this increase in moduli may be related to the relative loosening of the protein structure owing to S-S cleavage during homogenization and the easy exposure of free SH groups in the molecules to some extent. Amino acid residues in different regions of the same or different peptide chains are exposed, converged, and folded after the HPH process, which accelerates the formation of a stable spatial topology network [35]. According to Bi et al. [22], the strength of soy protein emulsion gels, as measured by their moduli values, increased when higher homogenization pressures were applied. This effect was attributed to the enhanced exposure of hydrophobic groups, resulting from the reduction in size of both protein chains and oil droplets within the mixed system as homogenization pressure intensified. In a study conducted by Baskinci & Gul [16], it was reported that the particle size decreased significantly with pressure applied to sesame protein up to 100 MPa, while the surface hydrophobicity increased. Additionally, Sun et al. [23] linked the enhanced gelation characteristics of protein isolates to the adequate energy supplied by HPH. This energy enables the restructuring of native proteins, exposes active sites, and facilitates protein-protein cross-linking.

The alteration in rheological properties can also be understood through the loss factor (G″/G′ = tan δ). Researchers have noted that tan δ represents the comparative distribution of “viscosity” to “elasticity” during the protein gel matrix formation process. A higher tan δ value corresponds to reduced elasticity in the gel system [66]. As shown in Figure 6D, there was little change in the tan δ value in the frequency range of 0–1 rad/s, and tan δ exhibited a rapidly increasing trend as the frequency increased above 1 rad/s. However, tan δ was detected below 1 throughout the frequency range in all samples, indicating that the samples exhibited solid-like elastic behavior [59]. The highest tan δ values (0.13–0.84) were detected in the control sample at almost all frequencies. HPH pretreatment of the hazelnut protein suspension caused a decrease in the tan δ values of the gel samples, which was especially evident at high frequencies (10 rad/s). The results indicate that the control sample had relatively weak bulk elasticity. The lowest tan δ value (0.37) at the highest frequency was detected in the gel sample pretreated at 100 MPa, followed by the sample pretreated at 150 MPa (0.47). Similarly, Zheng et al. [59] reported that with the increase in the pressure applied to the soy protein solution, a decrease in the final tan δ values occurred, but there was a partial increase at higher pressures. Our results show that modification of hazelnut proteins by HPH can significantly increase the elasticity of gels compared to control hazelnut protein gels.

The Power law model (Equations (2) and (3)) was applied to the experimental frequency sweep test data and provided a good fit (*R*^2^ = 0.987–0.999) (Table 3). Various studies have revealed that the power law model is the best model for explaining the change in moduli against the frequency of protein gels [22,23,36]. The K′ and K″ values followed the trends of G′ and G″, which helped define the viscoelastic properties of the material. For the control sample, the K′ and K″ values were calculated to be 7.445 and 1.415, respectively, and increased depending on the homogenization pressure. The maximum values (29.861 and 5.909, respectively) were obtained for the gel sample pretreated at 150 MPa. The increase in K′ and K″ values in the protein gels due to HPH application shows that the resulting gel is more flexible and has higher viscous properties. Findings regarding the increase in K′ and K″ values of the gels produced after homogenization pretreatment are included in the literature [24,36]. However, some studies have reported that K′ and K″ increase with the application of excessive pressure (>100 MPa). It has also been reported that these values decrease [23,25]. The other constants of the power law model are n′ and n″ (frequency exponents). When the power-law frequency exponent value approaches 0, the material behaves like a pure elastic gel, whereas a power-law exponent value approaching 1 indicates a pure viscous gel [67]. The n′ and n″ values calculated with the Power Law model were in the range of 0.074–0.146, indicating the presence of gels with weak physical properties. A significant decrease in the n′ and n″ values was observed because of the increased pressure applied to the hazelnut protein suspension before gelation (*p* <0.05). Comparable findings were noted in earlier research on sesame protein gel [24] and hazelnut beverage gel [25], where the control samples exhibited the highest *n′* and *n″* values, which subsequently decreased with HPH pretreatment. This indicates that the control sample’s elastic modulus values demonstrated greater frequency dependence compared to the gel samples that underwent homogenization pre-treatment. The observed reduction in n′ and n″ values as pressure increased suggests a diminishing frequency dependence of the elastic properties [22].

### 3.7. WHC and Gel Strength

**Figure 7:**
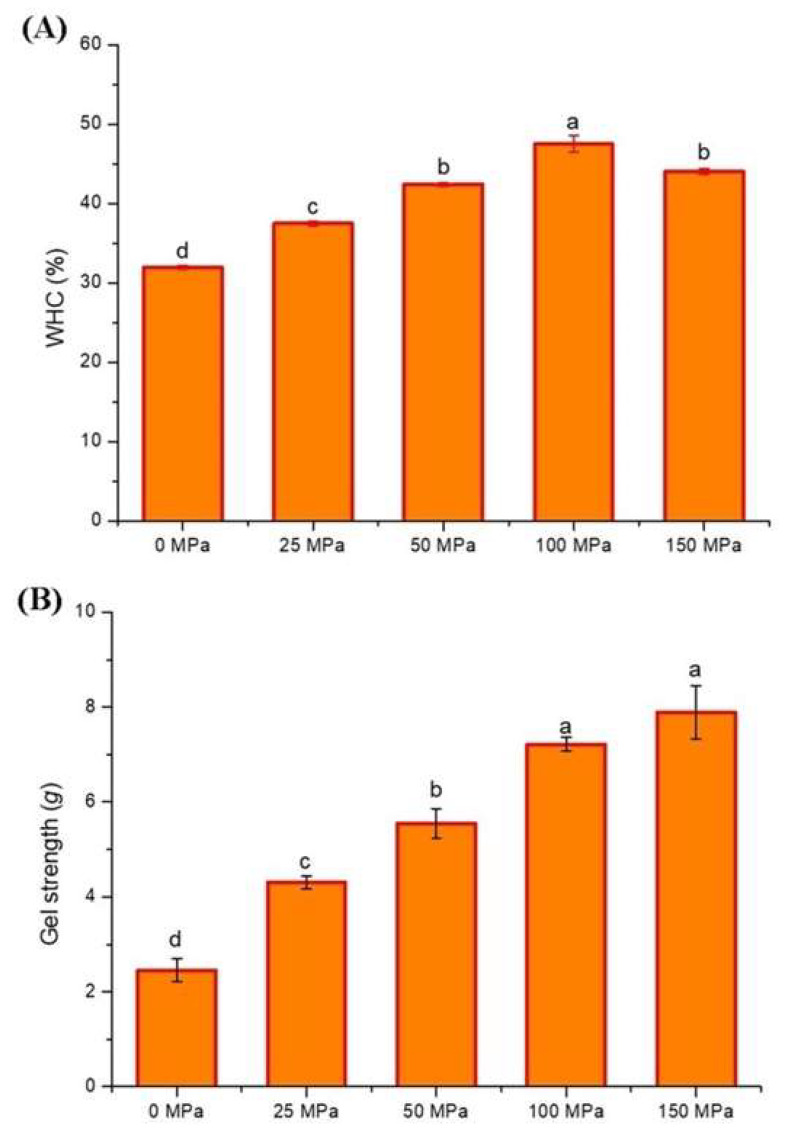
Water holding capacity (WHC) (**A**) and gel strength (**B**) of the acid-induced hazelnut protein isolate gels produced from HPH-treated hazelnut protein solution. (a–d lowercase letters refer to significant differences between the samples).

The water holding capacity (WHC) stands as a crucial functional attribute of gels, as the loss of water leads to gel contraction, alterations in texture, and unfavorable quality characteristics. The WHC results for hazelnut protein gels are shown in Figure 7A. The WHC of the control gel sample was determined to be 31.95%. HPH application before gelation significantly improved the WHC of the gels (*p* < 0.05). The highest WHC was determined in the hazelnut protein gel sample pretreated at 100 MPa, and higher-pressure application led to the weakening of the WHC. Similar results were obtained in our previous studies [24,25]. A significant increase in the WHC of the protein gel was achieved by increasing the pressure up to 100 MPa. In contrast, a decrease was detected with increasing pressure in the extremely high-pressure range. The increase in WHC can be attributed to the reduction in protein particle size resulting from HPH pretreatment. This size reduction leads to an expanded surface area, causing the protein’s molecular structure to unfold and altering its subunit and aggregation structure. The process exposes some hydrophobic groups, enhancing the hydrophobic effect, which facilitates gel formation [68]. Thus, a denser, more uniform, and stronger gel structure with smaller pores is formed, which can capture and hold water more effectively during centrifugation, thereby increasing the WHC [69]. Particle size is also important for WHC, and smaller particles facilitate water penetration and adhesion to protein matrices [70]. Xie et al. [53] observed a comparable outcome, noting that as pressure increased, the water holding capacity (WHC) of cod proteins significantly improved. This enhancement was linked to the reduction in particle size and the subsequent increase in protein surface area. Wu et al. [71] also reported that reducing proteins’ particle size facilitated the improvement of WHC. On the other hand, the decrease in WHC at 150 MPa may be attributed to the denaturation of protein due to excessively high pressure and destruction of the spatial structure of the protein. The decrease in the hydrophobicity of proteins and the increase in porosity and particle size also play a role in the formation of a protein gel network structure that is too weak to retain water [72,73]. If the homogenization pressure is high, insoluble aggregates are formed in the protein. An increase in aggregation and sedimentation reduces the water-holding capacity [74].

Gel strength, which represents the textural and structural compactness of the gels, is a crucial physical evaluation metric. Figure 7B shows the strength of the acid-induced hazelnut protein gels. The gel strength of the control sample was 2.46 g, which increased in parallel with the increase in pressure, and the highest value (7.89 g) was determined in the gel sample pretreated at 150 MPa. The reduction in protein particle size and increase in specific surface area due to increased pressure, and the increase in cross-linking between proteins and water molecules, resulting in the trapping/holding of water molecules in the protein and the formation of a more uniform network structure, explain the increase in gel strength [75]. Similar results were obtained in our previous study on the gelation of hazelnut beverages [25]. Zheng et al. [59] reported that the hardness of soy protein isolate gels showed an increasing trend because of HPH treatment, and this was probably due to the decrease in particle size of soluble aggregates in soy protein isolate with HPH. Moreover, HPH may cause partial opening and denaturation of proteins, which may increase interactions between proteins and increase hardness [59]. The high shear force during HPH disrupts the non-covalent interactions of proteins, exposing reactive groups such as free SH and hydrophobic groups [76,77]. These exposed groups lead to increased intermolecular interactions, which influence gel strength [77]. Research by Kang et al. [64] demonstrated that HPH treatment enhanced the solubility, free-SH content, and surface hydrophobicity of soy 11S globulin protein. This improvement in protein solubility and non-covalent binding facilitated gel formation, resulting in an enhanced gel structure. In another study, Wang et al. [78] attributed the enhanced gel strength of wheat gluten gel to several factors resulting from HPH treatment. These factors included the partial unfolding of the protein, the exposure of hydrophobic amino acid residues, and the increased hydrophobic interactions among protein molecules. It has been observed that applying a pressure higher than 100 MPa to hazelnut protein causes a decrease in gel strength. Excessive high-pressure application causes an increase in particle size in the protein solution and reduces protein solubility [11], resulting in lower protein-water interactions, which explains the decrease in gel strength.

### 3.8. Physical Stability

**Figure 8:**
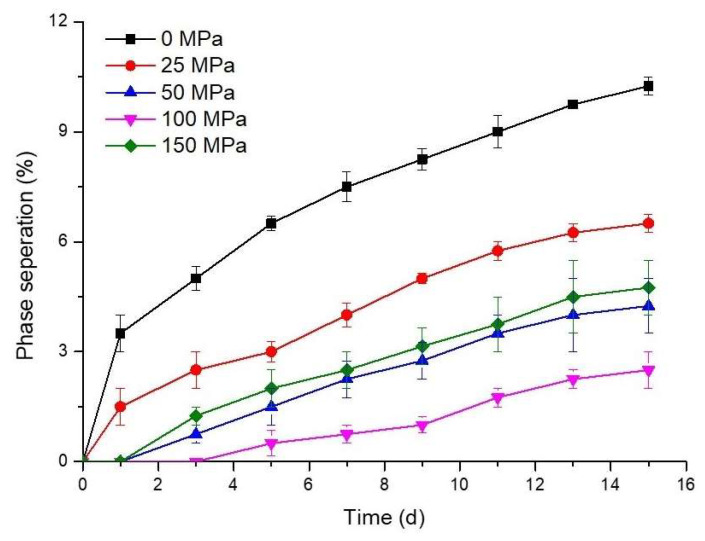
Phase separation of the acid-induced hazelnut protein isolate gels during storage.

The physical stability of GDL-induced hazelnut protein gels during storage was determined by considering phase separation. As shown in Figure 8, phase separation during storage was higher in the control gel sample than in the gel samples pretreated with HPH. Initial phase separation was observed in the control and 25 MPa pressure-treated gel samples on day 1, while it occurred on day 3 for the 50 and 150 MPa pressure-treated samples. The 100 MPa pressure-treated sample exhibited phase separation on day 5. The control sample demonstrated swift phase separation within the first 5 days of storage, followed by a subsequent reduction in the separation process. In hazelnut protein gels pretreated with HPH (except for the sample pretreated at 25 MPa pressure), phase separation was almost constant but at a low speed throughout storage. A similar trend was observed in sesame protein gels depending on the homogenization pressure [24]. At the end of storage (day 15), 10.25% phase separation was observed in the control sample, and a significant decrease in phase separation was observed with HPH pretreatment. The gel sample treated at 100 MPa exhibited minimal phase separation (2.53%), while a notable increase to 4.77% was observed when the pressure was raised to 150 MPa. Generally, higher pressures resulted in substantial improvements in phase separation, aligning with the water retention findings for hazelnut protein gels. This phenomenon is associated with the formation of a more compact gel structure and is also in line with the results from gel strength and rheological studies.

## 4. Conclusions

Texture is considered one of the most important characteristics for consumers when determining food quality, alongside nutrition, appearance, and taste. Gelation is a primary mechanism to create semi-solid and soft solid textures in food. Protein gels are of interest for food, biomedical, and pharmaceutical applications due to their high nutritional value, biocompatibility, and biodegradability. Additionally, improving the gelation properties of plant-based proteins, considered alternatives to animal-based proteins, has gained significance. Thus, the present study investigated the effect of HPH pretreatment on the acid-induced aggregation behavior of hazelnut protein isolates, and the effects of HPH at different pressures (0–150 MPa) on the structural, rheological, and gel properties of hazelnut protein gels were determined. The decrease in particle size with HPH pretreatment applied to the protein suspension contributed to obtaining a protein gel with high gel strength and WHC compared to the control group. The gelation mechanism of the protein was improved by HPH application, gelation time was shortened, and gels with high apparent viscosity and dominant viscoelastic properties were formed. The β-sheet content, which plays a vital role in gelation, tended to increase due to HPH application to hazelnut suspensions and played a role in improving the gel properties, which coincided with the gel strength and WHC. Hydrophobic interactions and disulfide bonds were the main intermolecular forces in the gelation of hazelnut protein with GDL, and increasing the pressure applied before gelation (up to 100 MPa) increased the dominant chemical bond content in the gels. The physical stability of the gels also improved. The results revealed that HPH pretreatment positively affected the gelling properties of the acid-induced hazelnut protein gels. However, further studies are required to investigate the effects of HPH on the gelation process, fully reveal its regulatory potential on protein networks, and apply protein gels with improved gel properties in the food systems. Objectives include improving plant-based acidified processing and products, utilization in a delivery system, and the development of vegan foods.

## Figures and Tables

**Table 1:** Acidification kinetic values of hazelnut protein with GDL after HPH treatment.

Samples	*V_max_*	*T_max_*	*T*_5.0_	*T*_4.5_
0 MPa	0.066 ± 0.006 ^b^	5.0 ± 0.0 ^a^	60.0 ± 0.0 ^a^	142.5 ± 3.53 ^a^
25 MPa	0.077 ± 0.009 ^ab^	5.0 ± 0.0 ^a^	52.5 ± 3.53 ^b^	127.5 ± 10.6 ^ab^
50 MPa	0.077 ± 0.009 ^ab^	5.0 ± 0.0 ^a^	52.5 ± 3.53 ^b^	122.5 ± 10.6 ^ab^
100 MPa	0.087 ± 0.001 ^ab^	5.0 ± 0.0 ^a^	50.0 ± 0.0 ^b^	112.5 ± 3.53 ^b^
150 MPa	0.09 ± 0.006 ^a^	5.0 ± 0.0 ^a^	50.0 ± 0.0 ^b^	115.00 ± 7.07 ^b^

Values are given as mean ± standard deviation. Different letters in the same column indicate significant differences between samples (*p* < 0.05). *V_max_*, Maximum acidification rate; *T_max_*, time to reach maximum acidification rate; *T*_5.0_, time to reach pH 5.0; *T*_4.5_, time to reach isoelectric point.

**Table 2:** The intermolecular forces of acid-induced hazelnut protein isolate gels produced from HPH treated hazelnut protein solution.

Samples	Ionic Bonds (mg/g)	Hydrogen Bonds (mg/g)	Hydrophobic Interactions (mg/g)	Disulfide Bonds (mg/g)
0 MPa	0.16 ± 0.03 ^c^	0.71 ± 0.01 ^d^	6.26 ± 0.09 ^c^	1.51 ± 0.03 ^c^
25 MPa	0.26 ± 0.02 ^b^	0.89 ± 0.01 ^c^	6.88 ± 0.11 ^b^	1.47 ± 0.06 ^c^
50 MPa	0.38 ± 0.01 ^a^	1.09 ± 0.03 ^b^	7.02 ± 0.16 ^b^	1.53 ± 0.08 ^c^
100 MPa	0.32 ± 0.02 ^a^	1.98 ± 0.07 ^a^	8.62 ± 0.21 ^a^	2.54 ± 0.09 ^b^
150 MPa	0.35 ± 0.04 ^a^	1.73 ± 0.02 ^a^	8.27 ± 0.33 ^a^	2.86 ± 0.03 ^a^

Values are means ± standard deviation. There appears to be a significant difference between samples of different letters (*p* < 0.05).

**Table 3:** Rheological parameters of acid-induced hazelnut protein isolate gels produced from HPH treated hazelnut protein solution.

Samples	$\eta_{\mathrm{app}}=K\times {\gamma ˙}^{n-1}$				$G\prime =K\prime \times \omega^{n\prime }$			$G^{\prime \prime }=K^{\prime \prime }\times \omega^{n^{\prime \prime }}$		
	Ƞ_50_ (mPas)	K	*n*	R^2^	K′	*n*′	R^2^′	K″	*n*″	R^2^″
0 MPa	16.288 ± 1.622 ^d^	0.169 ± 0.022 ^d^	0.412 ± 0.041 ^a^	0.9937	7.445 ± 0.517 ^d^	0.129 ± 0.007 ^a^	0.9881	1.415 ± 0.211 ^c^	0.146 ± 0.008 ^a^	0.9941
25 MPa	14.545 ± 0.936 ^d^	0.286 ± 0.018 ^c^	0.375 ± 0.019 ^a^	0.9936	17.178 ± 0.946 ^c^	0.127 ± 0.006 ^a^	0.9999	3.763 ± 0.508 ^b^	0.129 ± 0.011 ^a^	0.9953
50 MPa	26.23 ± 1.194 ^c^	0.346 ± 0.061 ^c^	0.348 ± 0.037 ^b^	0.9972	21.924 ± 1.076 ^b^	0.123 ± 0.004 ^c^	0.9943	4.068 ± 0.477 ^b^	0.101 ± 0.006 ^b^	0.9968
100 MPa	31.577 ± 0.992 ^b^	0.425 ± 0.039 ^b^	0.331 ± 0.047 ^b^	0.9975	24.347 ± 1.961 ^b^	0.119 ± 0.008 ^b^	0.9975	4.522 ± 0.606 ^b^	0.088 ± 0.005 ^bc^	0.9959
150 MPa	43.936 ± 1.044 ^a^	0.607 ± 0.055 ^a^	0.321 ± 0.034 ^b^	0.9915	29.861 ± 2.089 ^a^	0.118 ± 0.006 ^b^	0.9964	5.909 ± 0.427 ^a^	0.074 ± 0.018 ^c^	0.9874

Values are means ± standard deviation. There appears to be a significant difference between samples of different letters (*p* < 0.05). K, Consistency index; *n*, flow behavior index; K′ and K″, Power law model constants; n′ and n″, frequency exponents; R^2^, the degree of model fitting.

## Data Availability

The original contributions presented in the study are included in the article. Further inquiries can be directed to the corresponding authors.
